# Prevalence and Determinants of Occupational Injuries among Solid Waste Collectors of Zoomlion Ghana Limited

**DOI:** 10.1155/2021/6914529

**Published:** 2021-12-31

**Authors:** Patrick Ephraim, Judith Koryo Stephens, Gustavus A. Myers-Hansen, Richard Y. Otwey, Samuel Amon, Maxwell Kwasi Kporxah, Albert Abaka-Yawson

**Affiliations:** ^1^Department of Biological, Environmental and Occupational Health, University of Ghana, Accra, Ghana; ^2^Department of Epidemiology and Disease Control, University of Ghana, Accra, Ghana; ^3^Department of Nutrition and Food Science, University of Ghana, Accra, Ghana; ^4^Department of Medical Laboratory Sciences, University of Health and Allied Sciences, Ho, Ghana

## Abstract

**Background:**

Globally, occupational injuries account for 15% of the mortalities associated with occupational accidents. The work of the solid waste collectors exposes them to numerous occupational hazards, which results in injuries. Increasing rates of occupational injuries from 43.7% to 63.9% among solid waste collectors in sub-Saharan Africa opens room for more research to be done. The study assessed the magnitude of occupational injuries and associated factors among solid waste collectors of Zoomlion Ghana Limited in the Accra Metropolis.

**Methods:**

A cross-sectional quantitative study was carried out among the solid waste collectors. The occupational injuries and their associated factors among the solid waste collectors were assessed using questionnaires. Multistage sampling approach was used to select study respondents. Data were collected through the administration of questionnaires. Bivariate and multivariable logistic regression was used to assess the association between the dependent and independent variables.

**Results:**

In this study, 21.79% (78/358) with 95% CI (0.1749, 0.2608) among the solid waste collectors reported having at least one work-related injury in the last 6 months. The factors that were significantly associated with at least one occupational injury among the solid waste collectors in the Accra Metropolis were work duty (collection and transportation), the zone of assignment for respondents, and lack of personal protective equipment (PPE).

**Conclusion:**

This study showed that the prevalence of occupational injuries among municipal solid waste collectors in the Accra Metropolis was lower as compared to similar research conducted in Ethiopia, Egypt, and India. Working in the collection and transportation category and lack of PPE for use at work were significantly and positively associated with occupational injury among the solid waste collectors. Again, working in the La Dade Kotopon zone had reduced odds of sustaining injuries as compared to those in the Ablekuma South zone. The result of the study demonstrated that cuts/puncture was the injury that was mostly sustained by the municipal solid waste workers, while the leg was the body part that was mostly injured followed by the hands. Public health education in the municipality should target solid waste collectors with the aim of improving their health-seeking behaviour.

## 1. Background

Municipal solid waste (MSW) comprises several kinds of waste such as unwanted food items, discarded papers, broken glasses, used plastic materials, rubbers, and metals [1]. Unwanted paint containers and construction materials also form part of solid waste [2]. A wide variety of human activities results in the generation of household or municipal solid waste. Studies have indicated that about 55–80% of municipal solid waste is generated from households in developing countries. Commercial activities also account for 10–30% of the solid water, while industrial, institutional, streets, and other activities contribute to the remaining quota [3–5].

Waste that is generated from commercial, industrial, and household sources are extremely made up of different matter [5] and have varying physical features because of their origin. Miezah et al. [2] reported that the average waste generation per day in Ghana ranges from 0.2 to 0.8 kg/person irrespective of the person's social or economic status. Reports also indicate that several sub-Saharan African cities including Ghana have the same waste generation rate [7, 8]. However, the Organization for Economic Cooperation and Development (OECD) reported an increased waste generation rate of 1.39 kg/person/day [9]. In a study conducted in Nigeria by [10], their results showed that an average daily quantity of waste generated per person was 0.59 kg/day, while Daniel and Perinaz [11] also reported in their study that in low-income countries 0.74 kg of waste is generated per person/day.

Management of municipal solid waste is a burden to urban governance in most developing countries due to the increasing rate of solid waste generation and the impact it has on the environment [12, 13].

Solid waste management in Ghana includes storage, collection, transport and final disposal at landfill sites or sometimes to the waste recycling plant for recycling and composting processes [14]. Also, with the increasing technological advancement and industrial establishment in Ghana as a developing country, compost and recycling plants have been established that play an important role in waste management in Ghana especially in the capital city, Accra.

Generally, solid waste is dumped or littered directly on the ground. Municipal solid waste workers then use brooms to sweep and use shovels to gather them in waste bins. In some cases, plastic bags or baskets are used to keep the waste, and picking is done manually. With the help of a sack or pushcart, the waste collectors move the waste to collection points. The waste is then emptied into a refuse truck manually. The strenuous activity of solid waste management exposes the solid waste collectors (SWC) to diverse forms of health hazards [15], although waste collection contributes greatly to human health by reducing exposure to the risk of several infectious diseases.

Work-related injuries (WRIs) and sicknesses are caused by several factors and remain one of the vital concerns of public health to which stakeholders need to pay attention [16]. For example, Driscoll et al. [17] reported on the global burden of occupational injuries and sicknesses among developing and developed regions. They found out that the rates of occupational injury fatalities in the developing regions of the world was at least two to five times higher as compared to North America and Western Europe. The International Labour Organization (ILO) reported that 270 million accidents and diseases related to occupation occur every year. Globally, studies have shown an increased rate of work-related deaths and injuries. Injuries related to work account for an approximated number of 3,400,000 disabling injuries. On a daily basis, a fatal injury occurs every 2 hours, which results in disabling injury every 8 hours [18, 19].

Work-related accidents and injuries play a major role in human and economic costs. In 2007, the annual compensation cost for workers was $85 billion [20]. Four percent of the world's gross national product was lost due to occupational accidents and illness [21]. Work-related injuries and illnesses are of global public health concern. The incidence rate varies by the type of occupation with the management of solid waste ranking sixth among the hazardous occupations with a rate of 35.5 per 100,000, after fishing while loggers, pilots and flight engineers, iron and steel workers, ranchers, and farmers follow in ascending order [22]. It is important for employers of municipal solid waste workers to encourage good health and safety of their staff by putting in place measures that will facilitate efficient and safe solid waste management. Engineering and administrative controls should be adopted in addition to the provision and use of PPE [23]. Job rotation among work components, employee salary increment, regular training on health and safety, job-specific guidelines in relation to maximum production limits, and provision of new and improved working equipment such as new bags and containers that can be wheeled are measures expected to reduce the burden of occupational injuries among SWC. Specific periodic health surveillance (PHS) is also required for solid waste workers to detect early signs of ailments associated with their work and to keep an eye on work ability [24].

Scientific research on the health and safety of solid waste collectors have mostly focused on workers outside Ghana. This research seeks to determine the burden of occupational injuries and their associated socio-demographic, behavioural factors, as well as the working environment among solid waste collectors in Zoomlion Ghana Limited in the Accra Metropolitan Assembly.

Hypotheses are as follows:

  H1. Cuts/puncture will be the most prevalent frequent type of occupational injury recorded
  H2. Significant relationship between work environment and occupational injuries
  H3. Occupational injuries in Accra Metropolitan will be relatively lower than in other countries

### 1.1. Objectives of the Study

1. To determine the distribution of occupational injuries sustained by the solid waste collectors (SWC) of Zoomlion Ghana Limited in Accra metropolis
2. To assess the usage of personal protective equipment among SWC of Zoomlion Ghana Limited in the Accra metropolis
3. To determine behavioural characteristics associated with injuries among SWC of Zoomlion Ghana Limited in the Accra metropolis

## 2. Materials and Methods

### 2.1. Sampling Method

**Figure 1:**
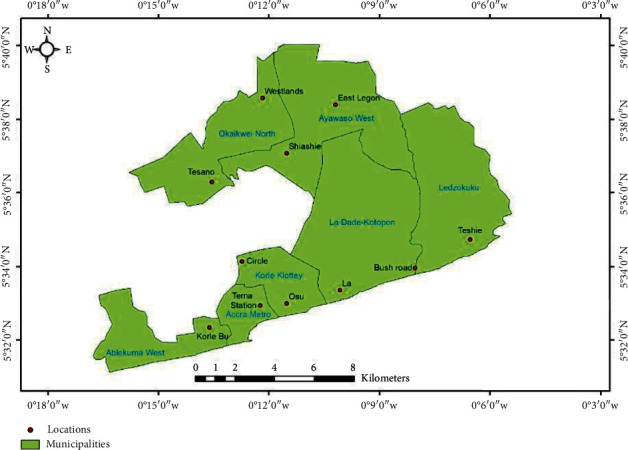
Map of the study area.

A cross-sectional survey was used in collecting the data. Zoomlion Ghana Limited in the Accra Metropolitan Assembly is divided into 11 zones. Each of the 11 zones within Accra Metropolis was considered a stratum Respondents for the study were selected using multistage sampling method. Computer-generated simple random technique was used to select 5 zones out of the 11 zones. The 5 zones included Ablekuma South, Ayawaso West, La Dade Kotopon, Teshie/Nungua, and Osu-Klottey. A proportion to size sample calculation was done to obtain the number of respondents needed to participate in the study from each of the zones. Each of these zones has a zonal head. Permission was obtained from the heads prior to data collection. This was done for zonal leaders and workers in the zone to be aware of the data collection that was to take place in their zones. Each zone has a specified day within the week that the workers meet after their duties. Map of the study area is shown in Figure 1.

### 2.2. Data Collection

Permission was sought from the management of Zoomlion Ghana Limited, Accra zone, for data collection. The data were collected over a 14 day period among the five zones that were selected in the metro using a simple random sampling technique. Data collection at each zone was done on a day that coincided with the zone's meeting day. On the data collection, the list of names of workers in the zones was obtained and numbers were assigned to them. Computer-generated simple random technique was used to select the respondents for the study. The next worker on the list after the worker who was previously selected randomly but did not meet the eligibility criteria was chosen to be part of the study (Table 1).

Zoomlion Ghana Limited in the Accra Metropolitan Assembly is divided into 11 zones. Participants for the study were selected using multistage sampling method. Each of the zones in the Metropolis was considered a stratum. Convenience sampling was used to select 5 (strata) zones out of the 11 zones in the metropolis.

The five zones or strata selected included Osu-Klottey, Teshie/Nungua, La Dade Kotopon, Ablekuma South, and Ayawaso West. After that, proportion to size sampling was used to determine the number of respondents from each zone to be involved in the study. In each of the five (5) zones (clusters), names of the workers in the zones were listed, and numbers were assigned to each employee. Computer-generated simple random technique was used to select the participants based on the number that was used for the study in that particular zone.

### 2.3. Interview

Explanation of the study was made plainly to all the respondents. Semistructured questionnaires (as shown in Appendix 1) were administered in the languages that were understood by each participant. The languages used for interviews were Ga, Twi, Ewe, and Fante. The questionnaire was translated into these languages based on the type that was well understood by the participant after their consent had been sought.

### 2.4. Ethical Approval and Consent

Ethical clearance was sought from the Ghana Health Services Ethical Review Committee (GHSERC). A letter of request was also used to seek permission from the management of Zoomlion Ghana Limited, Accra, for their employees to be used as the respondents of the study. Informed consent was obtained from the respondent who agreed to be part of the study by translating consent forms into various languages spoken by respondents to enable them to understand their role in their study.

### 2.5. Data Processing and Analysis

Each questionnaire was coded to avoid double entry of data and enhance verification. The principal investigator did data entry. Data were transferred from the questionnaire into Microsoft Excel software, 2013, and then exported to STATA software version 15.1 for analysis.

Descriptive statistics of the collected data was done for the variables in the study using statistical parameters: ranges, standard errors, means, standard deviation, percentages, as well as *p*-values. The result was presented in frequency tables. Bivariate analysis was done to check variables that are associated with the dependent variable individually. Multivariable logistic regression was then used to analyse variables that were found to have an association with the dependent variables.

The hypothesis testing used for the questionnaire of the study is chi-square test.

## 3. Results

### 3.1. Respondents' Demographic and Background Statistics

Table 2 presents detailed findings of the demographic data and background characteristics. A total of 358 municipal solid waste collectors took part in the study. Females accounted for a greater percentage of the respondents, 89% (317/358). The mean age for the respondents was 47.3 (±8.87) years with about 45% (160/358) being married. The educational levels of all respondents also ranged from the tertiary level to no formal education with most of them 83% (298/358) having worked with the company for more than 5 years.

### 3.2. Prevalence of Occupational Injuries

Table 3 shows the prevalence of occupational injuries among waste collectors understudied. A total of 21.79% (78/358) with 95% CI (0.1749, 0.2608) among solid waste collectors reported having at least one work-related injury in the last 6 months. Out of the 78 who had injuries, 74.36% (59/78) were injured once, while 24.36% (19/78) of them were injured twice or more. The highest number of occurrences of injuries was thrice in the past 6 months. The injuries ranged in severity from minor to severe injuries.

Injuries of the leg accounted for 46.15% (36/78) followed by hands and fingers 25.64% (20/78) and 17.95% (14/78), respectively. The sources of injuries were mainly due to falls 39.74% (31/78), while sharp objects accounted for 24.36% (19/78) of the injuries. The rest were due to collision, falling objects, hand tools, lifting of heavy objects, and snakebite. Loss of working days from the work-related injuries was reported to range from 1 to 30 days with 28.21% (22/78) losing less than 10 working days, while 8.97% (7/78) lost more than 10 working days.

About half, 52.56% (41/78), of the injuries were treated at a health facility, out of which 80.49% (33/41) were treated and discharged as outpatients, while 8.97% (7/41) of them were admitted for less than 10 days and 2.44% (1/41) was admitted for more than 10 days. The rest of the injuries, 46.15% (36/78) and 1.28% (1/78), were treated at home and by herbalists, respectively. Fifty-three (67.95%) out of 78 who reported occupational injuries had tetanus vaccination after the injuries.

**Figure 2:**
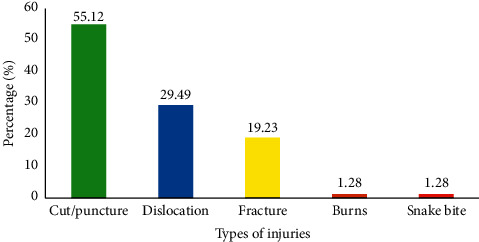
Type of occupational injury sustained by the solid waste collectors.

The common type of occupational injury, which was sustained by the respondents who reported occupational injuries within the past 6 months, was cuts (55.12%) followed by dislocation (29.49%), fracture (19.23%), and snakebite (Figure 2).

### 3.3. Distribution of Occupational Injuries among Solid Waste Collectors

The proportion of male workers who reported occupational injuries was 0.21 (17/41) and that of females was 0.78 (61/317). The difference in proportions is significant (*χ*^2^ = 10.52; *p* < 0.01). The age, marital status, and educational level were not significantly associated with occupational injury as shown in Table 4.

Occupational injuries were also significant among different work duties (*p* < 0.01) than work experience as well as the zone of assignment (*p* < 0.01), while the number of working days per week was not significantly associated with occupational injuries (Table 5).

### 3.4. Factors Associated with Occupational Injuries

Table 6 shows the association between predictor variables and occupational injuries was understudied. In the univariate analysis, males were 2.97 times more likely to sustain an injury when compared to females (COR = 2.97; 95% CI: 1.50, 5.87). Work duty was another variable, which was positively associated with occupational injury, those who were engaged in the collection and transporting of solid waste were 17.1 times more likely to be injured as compared to their counterparts (COR = 17.1; 95% CI: 3.21, 91.10). The likelihood of occupational injury was found to be higher (COR = 2.27; 95% CI: 1.31, 3.91) among workers who reported lacking PPE. The odds of sustaining occupational injury was 3.45 times higher among respondents who reported work stress (COR = 3.45; CI: 1.65, 7.24).

In the multivariable analysis, work duty (collection and transportation), the zone of assignment for respondents, and the lack of PPE were significantly associated with occupational injury among solid waste collectors after adjusting for all other socio-demographic, work-related, and behavioural factors. Among the respondents, those involved in collection and transportation were 8.5 times more likely to sustain an occupational injury (AOR = 8.5; 95% CI: 0.34, 48.81) than those involved in other work duties. The likelihood of occupational injury was found to be 2.24 more likely to occur among respondents who reported lack of PPE (AOR = 2.24; 95% CI: 1.21, 4.17).

## 4. Discussion

The response rate for the study was 92.03% (358/389) comprising 88.55% (317/358) females and 11.45% (41/358) males. This is consistent with other studies conducted on solid waste collectors [24–27]. This finding could be attributed to the fact that most of the respondents are of low education level that does not pave way for them to be employed in lucrative businesses, hence resorting to waste collection to earn a living [28].

About 45% (162/358) of the respondents have had education up to the junior high school level. Such a finding is similar to other studies, which showed that individuals involved in solid waste collection are of very low level of formal education [15, 24, 25]. Most of the respondents (96%) were between the ages of 30 and 65. This is consistent with findings from similar studies conducted on solid waste collectors [26, 27, 29]. This could be associated with the fact that old people involved in the solid waste collection especially the sweepers and collectors may not be in the capacity to be employed in high labour-intensive industries, hence finding themselves picking up waste to obtain income for daily living [28].

The overall prevalence rate of occupational injuries within the past 6 months was 21.79% (78/358). However, the result from this study is lower as compared to similar studies conducted in and outside of Africa that reported occupational injury prevalence of 73.2%, 63.9%, 43.7%, and 33.4%, respectively [24, 26, 27, 29]. The difference in the findings could be due to the fact most of the respondents in this study have worked as solid waste collectors for more than 6 years; hence, they have gained experience on hazards associated with their work and ways to avoid injuries. This is in accordance with other studies that have proven that more experienced waste collectors work safer, hence reducing the occurrence of occupational injuries [24, 30].

About 74% (59/78) of the respondents indicated that they have had injuries once during the past 6 months from this study that is in line with a study where the majority of the workers reported one occupational injury within a period of 6 months [27]. However, in a study conducted by Eskezia et al. [24], more than half of the respondents reported occupational injury for more than one time.

The most common type of injuries recorded in this study were cut/puncture and fracture that is in line with most research works done in Africa and Europe that showed cuts, punctures, and fractures as the most common injuries [1, 26, 27]. This could be attributed to the nature of waste these workers have contact with which is mostly a mixture of waste from several sources, which may contain sharp and slender objects. Again, the mode of waste collection that mostly involves manual handling together with dim lighting and rains due to early morning duties exposes them as well to the sharps and slender objects [31].

The leg is the part of the body that was mostly injured accounting for 46% (36/78) of the injuries followed by hands and fingers with 25% (20/78) and 17% (14/78), respectively. Similar studies conducted in Ethiopia also reported high percentages of injuries on the hands, legs, and fingers [24, 26, 27]. This might be because most solid waste collectors manually collect waste and put it into the sacks and trucks using their hands that increase the probability of cuts and abrasions at the hands and legs [27]. The result of the study could also be due to lack or underutilization of PPE by respondents as most of the respondents indicated they do not use PPE all the time because of lack of PPE or discomfort in using them. Again, most of the PPE used by the respondents were below standard and not in good condition to effectively protect the workers from injuries.

Regarding the cause of injury, falls resulted in almost 39% (31/78) of the occupational injuries followed by sharp objects and collisions with 24% (19/78) and 12% (10/78), respectively.

Meanwhile, other studies such as Frumkin [32], Eskezia et al. [24], and Rushton [1] reported that hitting by falling objects and injury by hand tools were the most common sources of injury. The difference in this finding could be due to the safety shoes that are worn by the study respondents that most of them complained were uncomfortable to wear due to their weight, and therefore, they chose to wear slippers and canvas while at work. It may also be due to the use of other unsuitable and unimproved protective equipment such as gloves, overcoats, and respirators for each job category [26].

Concerning the number of workdays lost due to injury, 28% (22/78) of those who were injured stayed home for less than 10 days, while 8.9% (7/78) of them stayed home for more than 10 days. A study indicated by [33] that the severity of an occupational injury is directly associated with the number of days lost due to injury. Moreover, more than half (62%) of the workers who had injuries never stayed home due to injury. On the other hand, workers who lost more than 10 working days (73.8%) were more than those who lost less than 10 working days in a study conducted by Eskezia et al. [24]. This could be attributed to the fact that most of the injuries sustained by the respondents in this study were minor injuries.

In this study, about 50% (41/78) of the injured respondents had their injuries treated at a health facility followed by 46% (36/78) who were treated at home. More than half 76.9% (60/78) of the respondents indicated they paid their own medical bills. This could explain why most of them preferred treating their injuries in the home due to the cost of health care services in Ghana.

In this study, the occurrence of occupational injuries was significantly associated with the work category, which is the collection and transportation of solid waste. This can be attributed to the fact that workers in this category are those involved in repeated heavy physical activities such as lifting and carrying of waste into trucks and tricycles for transport to dump and landfill sites [27]. These workers are highly exposed to sharps and other hazardous agents, and lack of the use of unimproved protective equipment results in the high occurrence of injuries among them. On the other hand, findings from other studies showed that occupational injuries are associated with work experience and work-related stress [24, 30, 34].

Literature indicates that there is a strong relationship between the use of PPE and reduced work accident rates among solid waste collectors. This is because PPE minimizes exposure to several hazards in the workplace [23]. This study also indicated that workers who lack PPE were 2.24 times more likely to report occupational injury than workers who had PPE. This is consistent with findings from other studies [27, 35].

The risk of occupational injuries for workers who work within the La Dade Kotopon zone was reduced by 84% as compared to those who work in Ablekuma South (AOR = 0.16; 95% CI: 0.06, 0.47).

These findings could be attributed to high alcohol consumption levels among workers in the Ablekuma South zone. The result of the study indicated that the odds of consuming alcohol for those in Ablekuma South is 1.3 times higher than those in La Dade Kotopon zone. Several literature on occupational injuries and associated factors found alcohol consumption to be positively associated with the occurrence of injuries among workers [36–39].

## 5. Conclusion

This study showed that the prevalence of occupational injuries among municipal solid waste collectors in the Accra Metropolis was lower as compared to similar research conducted in Ethiopia, Egypt, and India.

Working in the collection and transportation category as a solid waste worker and lack of PPE for use at work were significantly and positively associated with at least one occupational injury among the solid waste collectors. Again, the zone of the assignment was associated with occupational injuries, as working in La Dade Kotopon zone had reduced odds of sustaining injuries as compared to those in the Ablekuma South zone.

The result of the study demonstrated that cuts/puncture was the injury that was mostly sustained by municipal solid waste workers, while the leg was the body part that was mostly injured followed by the hands.

The report from the study indicated that the majority of the workers had access to some of the basic PPE needed for solid waste collection. However, most of them do not always use all PPE while on duty. Many of the workers reported not using all PPE at work always due to lack of PPE or discomfort associated with using certain PPE such as safety boots, overcoats, and nose masks.

### 5.1. Recommendations

1. There is a need for a regular and timely supply of improved and standard PPE such as hand gloves respirators, suitable and comfortable safety boots, and safety goggles for solid waste collectors. More importantly, occupational health and safety specialist must ensure regular and effective usage of PPE among workers.
2. Regular and effective occupational health and safety training prior to and after employment to educate workers about work hazards and risks. Such training must create awareness about behavioural factors such as alcoholism that could result in occupational injuries.
3. Policies should be formulated and implemented regarding the treatment of injured workers to help minimize the economic burden of injury treatment on workers.

## Figures and Tables

**Table 1:** Number of samples collected from each zone.

Zone	Number of samples
La Dade Kotopon	66
Teshie-Nungua	70
Osu Klottey	77
Ayawaso west	66
Ablekuma South	79
*Total*	*358*

**Table 2:** Demographic and background statistics.

Variable	Frequency	Percentage (%)
*Sex*		
Female	317	88.55
Male	41	11.45

*Age group*		
>30 years	344	96.09
≤30 years	14	3.91

*Residence*		
Urban	200	55.87
Peri-urban	158	44.69

*Marital status*		
Married	160	44.69
Single	81	22.63
Widow/widower	63	17.60
Divorced	43	12.01
Separated	11	3.07

*Religion*		
Christian	328	91.88
Muslim	24	6.72
Other(s)	3	0.84
Traditionalist	2	0.56

*Educational status*		
Junior high school	162	45.25
Primary	94	26.26
No formal education	62	17.32
Senior high school	38	10.61
Tertiary	2	0.56

*Work experience*		
>5 years	298	83.24
2 to 3 years	26	7.26
6 months to 1 year	19	5.31
4 to 5 years	15	4.19

*Monthly salary*		
100 Ghana Cedi	325	90.78
150 Ghana Cedi	33	9.22

**Table 3:** Prevalence of occupational injuries among municipal solid waste collectors of Zoomlion Ghana Limited in the Accra Metropolitan Assembly, Accra.

Variable	Frequency	Percentage (%)
*Occupational injury*		
Yes	78	21.8
No	280	78.2
Total	358	100

*Frequency of occupational injury (n* *=* *78)*		
Number of times of injury		
Once	59	75.6
Two or more times	19	24.4
Total	78	100

*Body part injured*		
Leg	36	46.2
Hands	20	25.6
Finger	14	18.0
Back	7	8.7
Head	6	7.70
Knee	5	6.4
Toe	3	3.9
Total	78	100

*Source of injury*		
Falls	31	39.7
Injured by sharp object	19	24.4
Collision	10	12.8
Hit by falling object	9	11.5
Injured by hand tool	5	6.4
Lifting heavy object	2	2.6
Snakebite	1	1.3
Fire	1	1.3
Total	78	100.00

*Number of working days lost*		
Injured but no home stay	49	62.8
Injured but stayed home for <10 days	22	28.2
Injured and stayed home for >10 days	7	9.0
Total	78	100.00

*Place of injury treatment*		
Health facility	41	52.6
Home	36	46.2
Herbalist	1	1.3
Total	78	100.00

*Tetanus vaccination*		
Yes	53	68.0
No	25	32.1
Total	78	100.00

*Frequency of those treated at a health facility (n* *=* *41)*		
*Number of days of admission*		
Treated but no admission	33	80.5
Treated but admitted for <10 days	7	17.1
Treated and admitted for >10 days	1	2.4
Total	41	100.00

^*∗*^Descriptive analysis.

**Table 4:** Prevalence of occupational injuries in the past 6 Months by sociodemographic factors among solid waste collectors.

Variable	Occupational injures		*χ* ^2^	*p*-value
	No (*n* = 280)	Yes (*n* = 78)		
	*n* (%)	*n* (%)		
*Sex*				
Male	24 (8.57)	17 (21.79)	10.5192	0.001^*∗∗*^
Female	256 (91.43)	61 (78.20)		

*Age groups*				
≤30	12 (4.26)	2 (2.56)	0.4812	0.488
>30	268 (95.71)	76 (97.44)		

*Marital status*				
Single	64 (22.86)	17 (21.79)	1.6055	0.808
Married	127 (45.36)	33 (42.31)		
Divorced	35 (12.50)	8 (10.27)		
Widow/widower	46 (16.43)	17 (21.78)		
Separated	8 (2.86)	3 (3.85)		

*Education level*				
No formal education	48 (17.14)	14 (17.95)	1.784	0.881
Primary	71 (25.36)	23 (29.49)		
Junior high school	129 (46.07)	33 (42.31)		
Senior high school	30 (10.71)	8 (10.27)		
Tertiary	2 (0.7)	0 (0.00)		

Notes. ^*∗*^—significant, *p* < 0.05; ^*∗∗*^—significant, *p* < 0.01; *N* = 358.

**Table 5:** Prevalence of occupational injuries in the past 6 months by working and behavioural factors among solid waste collectors of Zoomlion Ghana Limited in the Accra Metropolitan Assembly, Accra.

Variable	Occupational injures		*χ* ^2^	*p*-value
	No (*n* = 280)	Yes (*n* = 78)		
	*n* (%)	*n* (%)		
*Work experience*				
6 months to 1 year	17 (6.07)	2 (2.56)	2.8713	0.412
2 to 3 years	18 (6.43)	8 (10.26)		
4 to 4 years	11 (3.93)	4 (5.13)		
More than 5 years	234 (83.57)	64 (82.05)		

*Number of working days per week*				
<5 days (32 hours)	26 (9.26)	7 (8.97)	0.0181	0.991
5 days (40 hours)	127 (43.36)	36 (46.15)		
>5 days (48 hours)	127 (43.36)	35 (44.87)		

*Work duties*				
Collection	22 (7.86)	2 (2.56)	25.1988	0.000^*∗∗*^
Sweeping	13 (4.64)	1 (1.28)		
Collection and transportation	9 (3.214)	14 (17.95)		
Sweeping and collection	236 (84.29)	61 (78.21)		

*Zone*				
Ablekuma South	48 (17.14)	31 (39.74)	33.5663	0.000^*∗∗*^
Ayawaso West	46 (16.43)	20 (25.64)		
La Dade Kotopon	61 (21.79)	5 (6.41)		
Osu Klottey	60 (21.43)	17 (21.79)		
Teshie Nungua	65 (23.21)	5 (6.41)		

*Use of PPE at work*				
Sometimes	227 (81.07)	67 (23.93)	0.9678	0.325
Always	53 (67.95)	11 (14.10)		

*Reason for non-use of PPE*				
Lack of PPE	148 (52.86)	56 (71.79)	8.9257	0.003^*∗∗*^
PPE not comfortable	132 (47.14)	22 (28.21)		

*OHS training before employment*				
Yes	261 (93.21)	75 (96.15)	0.914	0.339
No	19 (24.36)	3 (3.85)		

*Periodic OHS training*				
Yes	207 (73.93)	70 (89.74)	8.7159	0.003^*∗∗*^
No	73 (19.03)	8 (10.26)		

*Satisfied with work*				
Yes	16 (5.71)	8 (10.26)	2.0123	0.156
No	264 (94.29)	70 (89.74)		

*Difficulty in sleeping*				
Yes	228 (81.43)	72 (92.31)	5.3183	0.021^*∗*^
No	52 (18.57)	6 (7.69)		

*Work-related stress*				
Yes	193 (68.92)	69 (88.46)	11.8604	0.001^*∗∗*^
No	87 (31.07)	9 (11.54)		

*Alcohol use*				
No	239 (85.36)	62 (79.49)	1.5702	0.21
Yes	41 (14.64)	16 (20.51)		

*Cigarette use*				
No	278 (99.29)	77 (98.72)	2367	0.627
Yes	2 (0.71)	1 (1.28)		

Notes. ^*∗*^—significant, *p* < 0.05; ^*∗∗*^—significant, *p* < 0.01; *N* = 358.

**Table 6:** Association between general characteristics and occupational injuries.

Variable	Occupational injures		Crude odd	Adjusted odd
	No (*n* = 280)	Yes (*n* = 78)	Ratio with 95% CI	Ratio with 95% CI
	*n* (%)	*n* (%)		
*Sex*				
Male	17	24	2.97 (1.50, 5.87)	2.2 (0.50, 10.22)
Female	31	256	1	1

*Work duties*				
Collection and transporting	14	9	17.1 (3.21, 91.10)	8.50 (1.34, 48.81)^*∗*^
Collection	2	22	1	1

*Zone*				
La Dade Kotopon	5	61	0.13 (0.05, 0.35)	0.16 (0.05, 0.47)^*∗*^
Teshie-Nungua	5	65	0.12 (0.04, 0.33)	0.31 (0.06, 0.52)
Ayawaso West	46	20	0.67 (0.33, 1.35)	1.84 (0.34, 1.55)
Osu Klottey	17	60	0.44 (0.22, 0.89)	0.05 (0.25, 1.21)
Ablekuma South	31	20	1	1

*Reason for no PPE use*				
Lack of PPE	56	148	2.27 (1.31, 3.91)	2.24 (1.21, 4.17)^*∗*^
PPE not comfortable	22	132	1	1

*Difficulty in sleeping*				
Yes	72	228	2.73 (1.12, 6.64)	2.16 (0.82, 5.71)
No	6	52	1	1

*Work stress*				
Yes	69	193	3.45 (1.65, 7.24)	1.30 (0.55, 3.10)
No	9	87	1	1

NB: ^*∗*^ denotes *p* < 0.05.

## Data Availability

Data are available upon request.

## Supplementary Materials

Supplementary Materials Semistructured questionnaires shown in Appendix 1 were administered in the languages that were understood by each participant.
