# Supplementary material for: Prevalence and Determinants of Occupational Injuries among Solid Waste Collectors of Zoomlion Ghana Limited
Source: J Environ Public Health. 2021 Dec 31;2021:6914529. doi: 10.1155/2021/6914529 (PMC8741403; doi:10.1155/2021/6914529)
Supplement: Supplementary Materials — Semistructured questionnaires shown in Appendix 1 were administered in the languages that were understood by each participant. [file 6914529.f1.docx]

| 1. **Questionnaire number** ………. **2. Date of interview DD/MM/YY**   ………/…...…../………..  Time …………. Interviewer name ……………………………………  Signature of Interviewer …………………… |
| --- |
| **PART A. DEMOGRAPHIC DATA** |
| **3. Age ………………………** |
| **4. Sex**  (Please tick as appropriate)  Male………… 0[ ] Female……… 1[ ] |
| **5. Place of Residence**  Urban ………………………1 [ ] Rural………………………..2 [ ] |
| **5. Marital Status** (Please tick as appropriate)  Single …………..……1[ ] Widowed/ Widower……..………4[ ]  Married………………2[ ] Separated …………………………5[ ]  Divorced……………..3[ ] |
| **6. Religion**  Christian……………………..1[ ] Muslim ……………………….2[ ]  Traditionalist………………...3[ ] Other………………………….4[ ] |
| **6. How do you pay for your medical care?**  National health Insurance ……………1[ ] Private Health Insurance………4[ ]  Self …..……………………………… 2[ ] Family………………………… 5[ ]  Paid by company……………………...3[ ] |
| **7. What is your highest educational level?**  No formal education ……………………… 1 [ ]  Primary……………………………………..2 [ ]  Junior High School…………………………3 [ ]  Senior High School…………………………4 [ ]  Tertiary………………………………………5[ ] |
| **8. Monthly Salary**  100-300 (GH¢) …………………1[ ] 400-600(GH¢) …………………….. 2[ ]  **>**600 (GH¢)……………………..3[ ] |
| **Part B (Work Environment)** |
| **8. How long have you been with your current company?**  6 months – 1 year………………1[ ] 4-5 years ………......................3[ ]  2-3 years ……………………….2[ ] > 5 years ……………………4[ ] |
| **9. What does your work entail? (Please tick as appropriate).**  Collection………………….1[ ] Sweeping ………………………2[ ]  Transporting ……………….3[ ] Collection & Sweeping ………..4[ ] |
| **10. How many days do you work per week?**  < 5 days (39 hrs) …………………… 1[ ]  ≥ 5 days (40 hrs) …………………….2[ ] Other ……………………….. 3[ ] |
| **11. Do you have Personal Protective Equipment (PPE)?**  Yes ……………………… 1[ ] No………………..2[ ]  **11 (b). Which of the following PPE do you use while at work?**  Eye googles ………………1[ ] Boots ……………..2 [ ] Nose cover .….3[ ]  Hand gloves ………………4[ ]  **11(c) How was the PPE obtained?**  Provided by company ……………….…1[ ] Provided by Employee …....2 [ ]  Other …………………………………...3[ ]  **11(d). Do you use Personal Protective Equipment while at work?**  Yes …………………….....1[ ] No ………………….2[ ]  **11(e). How often do you use your PPE while at work?**  Never …………………….. 1[ ] Always ………………………. 2 [ ]  Sometimes ……………….. 3[ ]  **11(f) What is the reason of not using Personal Protective Equipment?**  Lack of PPE …………………1[ ] Not Comfortable ……………………2[ ]  Not aware of PPE ……………3[ ] |
| **12(a). Job training on Occupational Health and Safety Prior to Employment.**  Yes …………………………….1 [ ] No …………………………..2 [ ]  **12(b). Periodic job training on Occupational Health and Safety Employment.**  Yes ……………………………… 1[ ] No ………………………….2 [ ] |
| **13. Are you Satisfied with your Job as a Solid waste worker?**  Yes …………………………1 [ ] No …………………………2 [ ] |

| **PART C. Behavioural Characteristics** |
| --- |
| **14. Do you have any difficulty with sleeping?**  Yes ……………………………….. 1[ ] No …………………….. 2[ ] |
| **15. Is your job as a solid waste worker stressful?**  Yes ………………………………...1[ ] No …………………………. 2[ ] |
| **16. Do you use substance?**  Yes ………………………………...1[ ] No ………………………. 2[ ]  **16(a). Do you drink alcohol?**  Yes ………………………………...1[ ] No ………………………. 2[ ]  **16(b). Do you smoke cigarrete?**  Yes ………………………………...1[ ] No ………………………. 2[ ] |
|  |

| **Part D-Work Related Injuries** |
| --- |
| **17. Have you had any injury related to your work within the past six months?**  Yes ……………………………...0[ ] No …………………………. 1[ ]  **17(a). If yes in 17, how many times have you sustained an injury(s)?**  Once ………………………1[ ] Two or more times …………...2[ ]  **17(b).Which of the following injury(s) were you affected with**? (**Please tick as appropriate)**.  Cut/ puncture ………………………1[ ] Abrasion ………………………2[ ]  Dislocation ………….……………. 3[ ] Fracture ………………………4[ ]  Eye injury ………………………… 5[ ] Ear injury……………………...6[ ]  Burn…………………………...……7[ ]  Others ………………………….…8[ ]  **17 (c). Which part(s) of the body is/was the injury?**  Hand ………………………………1[ ] Finger ………………………….. 2[ ]  Leg ………………………………...3[ ] Back ……………………………. 4[ ]  Knee………………………………..5[ ] Toe ……………………………... 6[ ]  Eye ………………………………... 7[ ] Tooth …………………...………8 [ ]  Head ……………………………….9[ ] Other ……………………….  **17(d). What was the source of the injury?**  Hit by falling object(s) ……………1[ ] Injured by hand tool …………………2[ ]  Falls………………………………3[ ] Lifting heavy object ………………....4[ ]  Splintering object ………………..5[ ] Collision ……………………………. 6[ ]  Other …………………………………..  **17(e). How many days did you stay home from work due the injury(s)**?  < 10 days ……………………………… 1[ ] ≥ 10 days ……………………… 2[ ]  **17(f) Where was the injury treated?**  At a health facility …………………….. 1[ ] At home …………………… 2[ ]  Herbalist ………………………………. 3[ ] Other ……………………….. 4[ ]  **17(g) If at a health facility, how many days were you admitted?**  < 10 days ……………………………… 1[ ] ≥ 10 days ……………………… 2[ ]  **18. Have you had tetanus vaccination?**  Yes ……………………………………1 [ ] No ………………………….... 2 [ ] |
